# Flexible Polymer Device Based on Parylene-C with Memory and Temperature Sensing Functionalities

**DOI:** 10.3390/polym9080310

**Published:** 2017-07-26

**Authors:** Min Lin, Qingyu Chen, Zongwei Wang, Yichen Fang, Jianfeng Liu, Yuchao Yang, Wei Wang, Yimao Cai, Ru Huang

**Affiliations:** 1 Institude of Microelectronics, Peking University, Beijing 100871, China; lin.min@pku.edu.cn (M.L.); chenqingyu@pku.edu.cn (Q.C.); wangzongwei@pku.edu.cn (Z.W.); fyczhdfx@pku.edu.cn (Y.F.); yuchaoyang@pku.edu.cn (Y.Y.); w.wang@pku.edu.cn (W.W.); ruhuang@pku.edu.cn (R.H.); 2 Innovation Center for Microelectronics and Integrated System, Peking University, Beijing 100871, China; 3 Department of Geriatric Cardiology of Chinese PLA General Hospital, Beijing 100871, China; shenlangliu@163.com

**Keywords:** wearable devices, flexible, resistive random-access memory, temperature sensor

## Abstract

Polychloro-para-xylylene (parylene-C) is a flexible and transparent polymer material which has excellent chemical stability and high biocompatibility. Here we demonstrate a polymer device based on single-component parylene-C with memory and temperature sensing functionalities. The device shows stable bipolar resistive switching behavior, remarkable storage window (>10^4^), and low operation voltages, exhibiting great potential for flexible resistive random-access memory (RRAM) applications. The I-V curves and conductive atomic force microscopy (CAFM) results verify the metallic filamentary-type switching mechanism based on the formation and dissolution of a metal bridge related to the redox reaction of the active metal electrode. In addition, due to the metallic properties of the low-resistance state (LRS) in the polymer device, the resistance in the LRS exhibits a nearly linear relationship at the temperature regime between 25 °C and 100 °C. With a temperature coefficient of resistance (TCR) of 2.136 × 10^−3^/°C, the device is also promising for the flexible temperature sensor applications.

## 1. Introduction

With the growing popularity of intelligent terminals, wearable devices are gradually changing our way of life. Wearable devices have a wide range of applications, including healthcare, smart home, communication, child custody, and so on. Generally, electronic devices used in wearable systems need to take into account several strict criteria. For example, such electronic devices should have superior mechanical flexibility and be able to be conformally attached to objects without affecting the performance of the devices. Furthermore, the weight and size of the devices should be keep small so as not to affect the user’s normal movements. In addition, when these devices are implanted in the body, safety must be adequately guaranteed and the cost should be acceptable to consumers. These applications require that the materials used for the wearable systems have good flexibility, light weight, and high biocompatibility.

Wearable systems are composed of multiple functional modules, such as memories [1,2], sensors [3,4], control and processing units [5,6], etc. In particular, as a medium for information storage, memory plays a vital role in wearable systems. In recent years, resistive random-access memory (RRAM) has emerged as a potential candidate for next-generation non-volatile memory because of its simple structure, excellent scalability, high switching speed, low power consumption, and high reliability [7,8,9,10,11]. Depending on the active materials, RRAM can be divided into organic RRAM and inorganic RRAM [12,13]. Among them, organic RRAM is very advantageous for wearable device applications due to its low cost, good flexibility, and simple structure [14,15,16,17,18]. Sensors, as a core component of wearable systems, have been extensively studied by industry and the scientific community. Particularly, temperature sensors are one of the parts of such devices raising the most concern because temperature has a close relationship with biological, chemical, physical and electronic systems. Wearable temperature sensors with flexible, light-weight, and implantable characteristics provide the possibility to monitor human body temperature, environmental conditions, and food safety [19,20,21,22,23]. In the past few decades, several types of flexible temperature sensors have been studied in depth, such as thermal resistance temperature sensors [24,25], thermocouple sensors [26,27], and organic field-effect transistors [28,29]. Bao et al. reported a flexible wireless temperature sensor based on Ni microparticle-filled binary polymer composites [30]. Fan et al. developed a flexible temperature sensor array based on a graphite-polydimethylsiloxane composite [31]. Although these temperature sensors have demonstrated their potential across diversified wearable applications, they show limited biocompatibility, especially in implantable systems.

Here, we demonstrate a polymer (organic) device based on polychloro-para-xylylene (parylene-C) with functionalities of memory and temperature sensing. Parylene-C is a flexible and transparent polymer material, which can be deposited by polymer chemical vapor deposition (CVD) with advantages of high step coverage, good purity without solvent contamination, and excellent compatibility with various substrates. In addition, parylene-C is a Food and Drug Administration (FDA)-approved and bio-friendly material, showing the prominent capability of being safely used within the human body. Therefore, it is very suitable for bio-friendly wearable device applications. Our flexible device exhibits very good storage characteristics with a large storage window, low operation voltages, and good stability. By analyzing the I-V curves and current images obtained by conductive atomic force microscopy (CAFM), we suggest that the metallic filamentary-type switching mechanism is a potential mechanism for the reversible switching behavior of the Al/parylene-C/W device. Due to the metallic properties of the low-resistance state (LRS) in the device, we further study the temperature sensing characteristics of the resistance in the LRS. After the set process, the resistance of the device is linearly related to temperature range from 25 °C to 100 °C, and its temperature coefficient of resistance reaches 2.136 × 10^−3^/°C. These results show that the polymer device can be used not only as a flexible memory device, but also as a temperature sensor. For wearable device applications, this versatile device with excellent biocompatibility and good flexibility can effectively reduce the fabricating cost and design complexity of wearable systems.

## 2. Materials and Methods

### 2.1. Device Preparation

**Figure 1:**
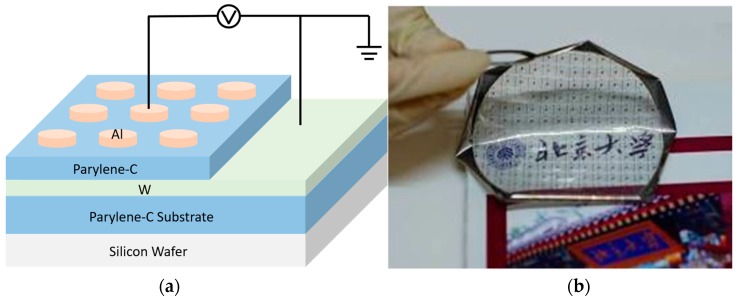
(**a**) Schematic of the fabricated flexible Al/ polychloro-para-xylylene (parylene-C)/W device; (**b**) Picture of the fabricated device with excellent flexibility and transparency.

As shown in Figure 1a, the polymer device has a metal/polymer/metal (MIM) sandwich structure. The fabrication process is as follows. First, about 5 um parylene-C (SCS, Indianapolis, Indiana, IN, USA) was deposited by polymer CVD on the silicon wafer as a flexible substrate. Then, W bottom electrode (WWW.ZNXC.CN, Beijing, China) with a thickness of 200 nm was deposited by direct-current (DC) sputtering followed by a lift-off process. Next, about 40-nm-thick parylene-C was deposited at room temperature. Then, the bottom electrode via was formed by photolithography and reactive ion etching. Finally, 200-nm-thick Al top electrode (WWW.ZNXC.CN, Beijing, China) was also formed by DC sputtering and a lift-off process. Without any chemical reagents, the as-fabricated device can be easily removed from the silicon wafer, as shown in Figure 1b. The samples without top electrodes for CAFM measurement were also fabricated using the same preparation method as above.

### 2.2. Characterization

All electrical characteristics of the polymer device were measured by Agilent B1500A (Keysight Technologies, Santa Rosa, California, CA, USA) in an ambient air environment. The bias was added to the top electrode with the bottom electrode grounded. To prevent the electrical breakdown of the fabricated device, a current compliance was set to 1 mA. The switching mechanism was analyzed by means of current maps using the CAFM system (Bruker Multimode 8, Santa Barbara, California, CA, USA). The current maps of the parylene-C/Pt structure were acquired by a surface scan under the CAFM scanning mode with a constant voltage. During the CAFM tests, the voltage was applied to the Pt bottom metal while the Pt/Ir-coated Si tip was grounded.

## 3. Results and Discussion

### 3.1. Resistive Switching Characteristics

**Figure 2:**
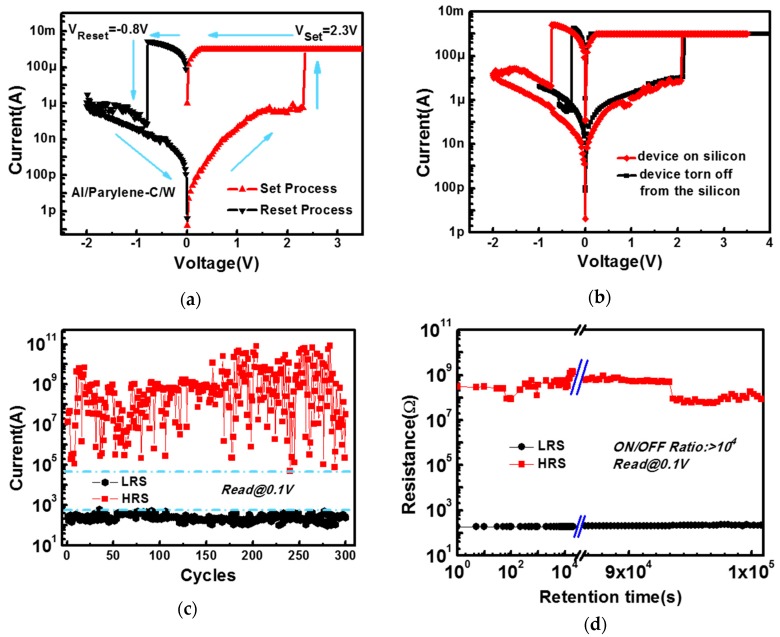
(**a**) Measured typical I-V curves of the Al/polychloro-para-xylylene (parylene-C)/W device; the arrows show the direction of voltage sweep; (**b**) The I-V characteristics of the fabricated device. The red diamonds show the I-V curve of the device fabricated on the silicon wafer, and the black squares show the I-V curve after the device was torn off from the silicon wafer; (**c**) Measured endurance characteristics of the polymer device by the direct-current (DC) sweep operation; (**d**) Retention behavior of the polymer device measured by applying a 0.1 V read voltage.

The memory behavior-based resistive switching of the Al/parylene-C/W polymer device on a flexible substrate is characterized, as shown in Figure 2. Figure 2a gives the measured typical I-V characteristics of the device with an area of 2 × 2 um^2^. The as-fabricated device is initially in a high-resistance state (HRS) and exhibits forming-free bipolar resistance characteristics. The device is switched to an LRS at about 2.3 V and switches back to an HRS at about −0.8 V. The device shows a high storage window value (>10^4^) with the ratio of high resistance in HRS to low resistance in LRS. The device prepared on a ~5 um flexible parylene-C substrate can be easily torn off from the silicon wafer with excellent flexibility and transparency. Then, the flexible device can be attached to any flat, curved, rigid, or soft surface. After being torn off from the silicon wafer, the electrical characteristics of the device do not change significantly, demonstrating the superior mechanical robustness and electrical stability of the materials, as shown in Figure 2b. To further evaluate the operational reliability of the flexible Al/parylene-C/W polymer device, memory performance characteristics such as endurance and retention are analyzed. The switching reproducibility of the polymer device is tested by a “write-read-erase-read” sequence, as shown in Figure 2c. The polymer device can be operated successfully for more than 300 cycles under DC sweep with an acceptable storage window value. As shown in Figure 2d, the polymer device also exhibits good retention properties for both LRS and HRS, showing >10^4^ storage window value over 10^5^ s. These results show the feasibility of the polymer device as a reliable nonvolatile polymer memory.

### 3.2. Resistive Switching Mechanism

**Figure 3:**
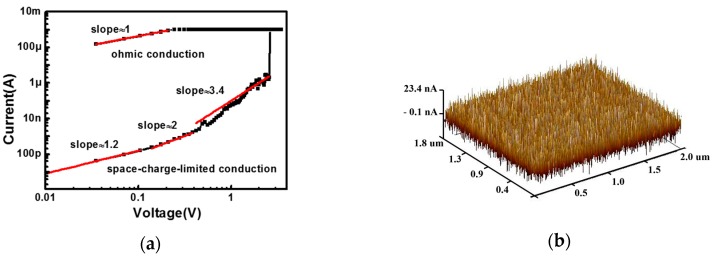
(**a**) The I-V curve with log-log scale from the set process. The low-resistance state (LRS) follows an ohmic conduction and the high-resistance state (HRS) follows a space-charge limited conduction (SCLC); (**b**) Current map of the fabricated conductive atomic force microscopy (CAFM) samples with an inert Pt electrode.

In order to analyze the resistive switching mechanism, a log-log plot of the I-V analysis is obtained in Figure 3a. It is worth noting that in the HRS there are three distinct regions correlated to the sweep voltages. At a low voltage region below 0.1 V, the current is approximately proportional to the voltage, which can be explained by thermally generated free carriers. At a voltage region between 0.1 and 0.5 V, there is a linear relationship between I and V^2^, showing a space-charge limited current. At a voltage region between 0.5 and 2 V, the slope of the I-V curve continues to increase. In this voltage region, the trap states in active layer are completely filled with injected carriers and thus the current increases rapidly. However, in the LRS, the current is linearly related to the voltage, showing an ohmic property, which corresponds to filamentary conduction induced by the formation of a conductive path connecting the top and bottom electrode. In order to further confirm the metallic filamentary-type switching mechanism, the CAFM testing method is introduced in this work. In this measurement, the CAFM samples was fabricated with an inert Pt electrode rather than an active electrode (Al or Cu). As shown in Figure 3b, the current map obtained in the tip/parylene-C/Pt structure under CAFM scanning mode shows a low current state with an average current value below 10 nA, even when a voltage of 10 V is applied to the sample (the highest voltage the CAFM system could supply). These results indicate that the active electrode plays a critical role in the resistive switching of the device and the polymer itself has no resistive switching behavior.

**Figure 4:**
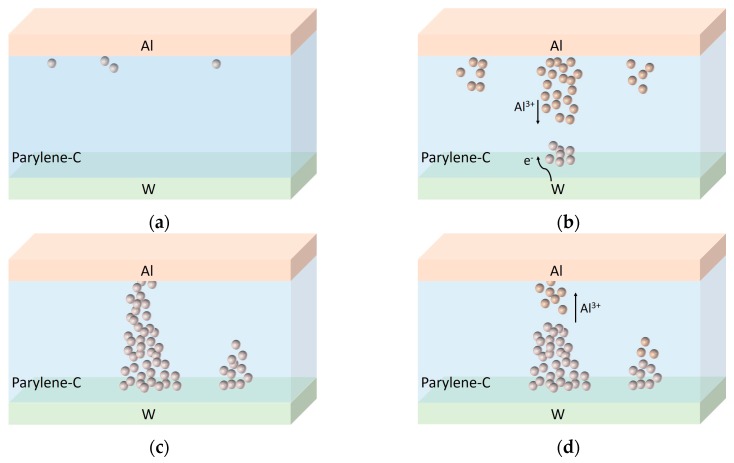
Schematic diagram of switching mechanism explaining the operation of the Al/polychloro-para-xylylene (parylene-C)/W device; (**a**) Initial low current state of the as-fabricated device; (**b**) Oxidation of Al at the top electrode, the migration of the Al ions into the parylene-C and the reduction of Al ions at the bottom electrode; (**c**) Formation of an Al conductive filament connecting the top and bottom electrodes; (**d**) Dissolution of the Al conductive filament at the weakest position of the filamentary path.

Based on the experimental results and theoretical analysis, we proposed a conduction model to illustrate the switching mechanism of the device, which is based on the formation and dissolution of a metal bridge related to redox reaction of the active metal electrode, as shown in Figure 4. Due to the non-conductive nature of parylene-C, the as-fabricated device is initially in an HRS (Figure 4a). When a positive voltage is applied to the Al top electrode, Al tends to be oxidized and the resulting Al ions migrate into the parylene-C layer under the action of the electric field. The subsequent reduction of Al ions and accumulation of Al atoms occur at the bottom electrode to form a metallic conductive filament connecting the top and bottom electrodes, and thus the device is switched to an LRS (Figure 4c). When the polarity of the applied voltage is reversed, the electrochemical dissolution of the Al conductive filament occurs and hence the device is switched back to an HRS (Figure 4d). It is widely believed that the rupture of the metallic conductive filament occurs at the weakest position of the filamentary path.

### 3.3. Temperature Sensing Characteristics

**Figure 5:**
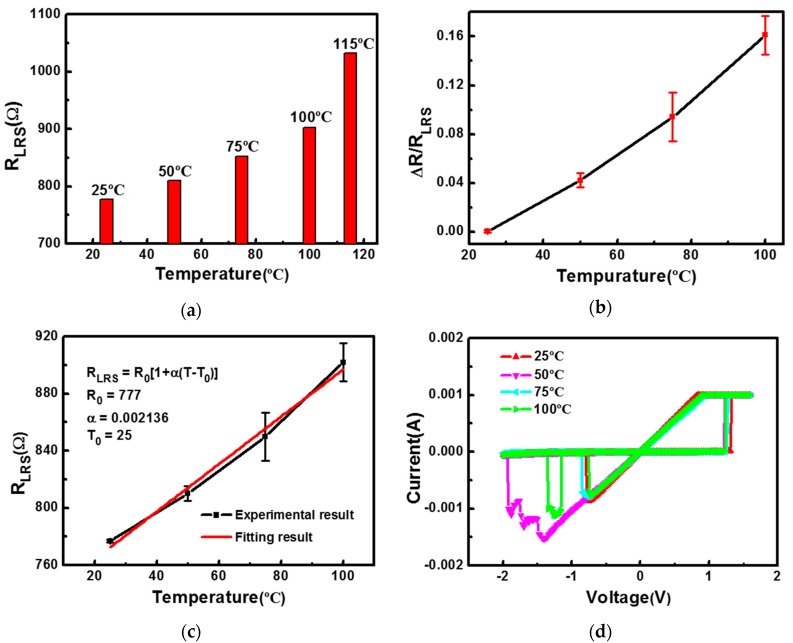
Temperature sensing characteristics of the polymer device; (**a**) Resistance of the low-resistance state (LRS) as a function of temperature ranging from 25 °C to 115 °C; (**b**) Resistance ratio of the LRS versus the temperature ranging from 25 °C to 100 °C; (**c**) The fitting result from the equation; (**d**) The I-V curves of the polymer device at different temperatures.

As shown in Figure 5a, since the conductive filament is composed of Al atoms, the lower resistance of the device increases with the increasing temperature. In particular, the lower resistance of the device varies with temperature in a linear manner approximately from 25 °C to 100 °C, which is consistent with the metallic properties of the conductive filament. However, at a temperature regime above 100 °C, the lower resistance of the device deviates from a linear change and exhibits a greater change, which is possibly partially due to the diffusion of Al atoms in the filamentary path at higher temperatures. The diffusion of Al atoms makes the conductive filament thinner, resulting in greater resistance changes at the same temperature change.

Due to the linear dependence of lower resistance on the temperature ranging from 25 °C to 100 °C, this flexible polymer device can be used as temperature sensor in addition to being used as a memory element, especially as wearable or implantable human body temperature-sensing device because its sensing capability from 25 °C to 100 °C can well cover the range of temperature of the human body. Therefore, we carefully investigated the temperature sensing characteristics of the device in this temperature range. Figure 5b shows the resistance ratio as a function of temperature from 25 °C to 100 °C. The result shows that the resistance ratio is approximately linear with temperature. We can use the following formulation to express the relationship between the measured resistance and the temperature:

(1) $$R_{LRS}=R_{0}\left[1+\alpha \left(T-T_{0}\right)\right]$$

where *R*_0_ and *R_LRS_* are the resistance of the device at the reference temperature *T*_0_ and operational temperature *T*, and α is the temperature coefficient of resistance (TCR). The TCR of the device can be obtained by measuring the resistance variation with the increased temperature range from 25 °C to 100 °C. As shown in Figure 5c, the TCR of the device obtained from the fitting curve is about 2.136 × 10^−3^/°C, which is lower than the theoretical value of the Al wire (3.93 × 10^−3^~4.10 × 10^−3^/°C) [32]. This may be due to the fact that the temperature control of the test system was not ideal during the test, so the actual temperature of the Al conductive filament was lower than the ambient temperature. Therefore, *R_LRS_* in Equation (1) is smaller than the actual value, and thus α is also relatively small. This suggests that accurately controlling the temperature of the sample to be measured can reduce the test error. Al is chemically active and susceptible to corrosion and oxidation, so it is rarely used as the temperature sensing element. However, in our device, Al conductive filaments formed in parylene-C are protected from oxidation and chemical corrosion due to the superior passivation effect of parylene-C against moisture, chemicals, and solvents, thus showing good temperature sensing characteristics. In order to verify the stability of the device, Figure 5d shows the I-V curves of the device at different temperatures. The almost coincident curve shows the good thermal stability of the device.

## 4. Conclusions

In summary, we have successfully demonstrated a flexible and transparent polymer device based on parylene-C with memory and temperature sensing functionalities. This device exhibits advantages of low operation voltages, large storage window, and good flexibility. In addition, it can be fabricated by a fully CMOS (Complementary Metal Oxide Semiconductor)-compatible process. The switching mechanism of the device as a memory element is verified by the I-V curves and the CAFM results, and the formation and dissolution of a metal bridge related to the redox reaction of the active metal electrode is responsible for the resistive switching behavior. Finally, based on the metallic properties of the conductive filament in this device, the temperature sensing characteristics of the device are carefully investigated. With a TCR of 2.136 × 10^−3^/°C, the device is also promising for a human body temperature sensing application at the temperature regime between 25 °C and 100 °C.
